# Chemosensitivity of Anaplastic Thyroid Cancer Based on a Histoculture Drug Response Assay

**DOI:** 10.1155/2015/967286

**Published:** 2015-03-18

**Authors:** Takashi Uruno, Chie Masaki, Junko Akaishi, Kenichi Matsuzu, Akifumi Suzuki, Keiko Ohkuwa, Hiroshi Shibuya, Wataru Kitagawa, Mitsuji Nagahama, Kiminori Sugino, Koichi Ito

**Affiliations:** Department of Surgery, Ito Hospital, 4-3-6 Jingumae, Shibuya-ku, Tokyo 150-8308, Japan

## Abstract

The chemosensitivity of anaplastic thyroid cancer (ATC) to some cytotoxic agents was investigated by the histoculture drug response assay (HDRA). Thirty specimens from 22 patients with ATC were obtained from surgically resected subjects. The drugs tested were paclitaxel (PTX), docetaxel (DOC), adriamycin (ADM), nedaplatin (254-S), cisplatin (CDDP), carboplatin (CBDCA), etoposide (VP-16), 5-fluorouracil (5-FU), mitomycin C (MMC), and cyclophosphamide (CPA). PTX was the most effective agent, and 25 of 29 cases (86.2%) had high inhibition rates (IRs; over 70%), while DOC, another taxane, had lower IRs (median, 32.6%). 254-S had the second highest IR (median 68.1%), higher than other platins, CDDP (median 47.3%) and CBDCA (median 27.4%). The IR of 50% dose PTX (20 *μ*g/mL, median 30.6%) was markedly decreased, while that of 50% dose 254-S (10 *μ*g/mL, median 63.3%) still retained its inhibition effect compared to 100% dose. Most recurrent samples had higher IRs than primary lesions, but the IRs of different drugs differed between primary and recurrent lesions, even with samples from the same patients. PTX has a higher IR to ATC tissues in the HDRA, which suggests that it may be a key drug for the treatment of patients with ATC.

## 1. Introduction

Anaplastic thyroid cancer (ATC) accounts for only 1 to 2% of all thyroid cancers, but it has the worst prognosis of any cancer, with a median survival of 5 months and a 1-year survival rate of 20% [1]. Cause-specific survival rates (CSS) differ significantly by UICC stage, with 6-month CSS of 60% for stage IVA, 45% for IVB, and 19% for IVC in Japan [2]. Some small studies have shown favorable results with aggressive surgery and adjuvant radiation and chemotherapy, but chemotherapy itself appears to have a limited effect on survival in most studies. The American Thyroid Association guideline [3] introduced regimens for ATC including paclitaxel, docetaxel, doxorubicin, cisplatin, and carboplatin, but there are no systemic therapies of proven benefit in terms of improved survival in advanced ATC [3]. On the other hand, in a trial conducted by the Eastern Cooperative Oncology Group from 1976 to 1982, the median survival of 39 patients with ATC was only 2.7 months, but two responses to doxorubicin plus cisplatin were durable, at 41.3 and 34.7 months, suggesting a possible impact on survival in selected patients with ATC [4].

The histoculture drug response assay (HDRA) is a representative in vitro drug-response assay method used for anticancer agents. Several clinical studies have shown that inhibition rates obtained using the HDRA can predict clinical responses to chemotherapy in patients with lung, breast, uterus, and other malignancies [5–14]. However, there are no reports about thyroid cancer, especially ATC. Therefore, the chemosensitivities of ATC to some major cytotoxic agents were investigated to identify more promising candidates that should be included in new chemotherapeutic regimens.

## 2. Materials and Methods

### 2.1. Subjects

Thirty specimens from 22 patients with ATC were obtained from surgically resected subjects. The specimens consisted of 18 primary lesions, 8 cervical local recurrences, 2 lung metastases, 1 axillary lymph node metastasis, and 1 chest wall metastasis. Nineteen of 30 samples had been treated by weekly paclitaxel (80 mg/m^2^) preoperatively. All specimens were histologically confirmed as ATC or ATC metastasis.

### 2.2. HDRA Procedure

Surgical specimens were sampled immediately from patients undergoing surgery for ATC. Fresh tumor tissue samples were obtained from each patient and transported to the laboratory at 4°C in Hank's balanced salt solution (HBSS).

Methods for the HDRA were as reported by Furukawa and colleagues [5] and commercially measureable (SRL Inc., Tokyo, Japan).

The cancerous portions of the specimens were minced into pieces approximately 10 mg in weight, which were then placed on collagen sponge gels manufactured from pigskin in 24-well microplates. The plates were incubated for 7 days at 37°C in the presence of drugs dissolved with RPMI 1640 medium containing 20% fetal calf serum and left in a humidified atmosphere containing 95% air-5% CO_2_. The concentrations of drugs were 40 and 20 *μ*g/mL for paclitaxel (PTX), 100 *μ*g/mL for docetaxel (DOC), 15 *μ*g/mL for adriamycin (ADM), 20 and 10 *μ*g/mL for nedaplatin (254-S), 20 *μ*g/mL for cisplatin (CDDP), 25 *μ*g/mL for carboplatin (CBDCA), 500 *μ*g/mL for etoposide (VP-16), 300 *μ*g/mL for 5-fluorouracil (5-FU), 2 *μ*g/mL for mitomycin C (MMC), and 30 *μ*g/mL for cyclophosphamide (CPA). These concentrations of each drug were determined based on experience with colon, gastric, lung, and breast cancers. Quadruplicate samples were used for each setting. After histoculture, 100 mL HBSS containing 0.1 mg/mL type I collagenase and 100 mL MTT solution, dissolved in 5 mg/mL phosphate buffer solution, were added to each culture well and incubated for another 16 hours. Following extraction with dimethyl sulfoxide, absorbance of the solution in each well was read at 540 nm. Absorbance per g of cultured tumor tissue (optical density/weight: OD/*W*) was calculated from the mean absorbance of tissue from 4 culture wells and the tumor tissue weight was determined prior to culture.

The inhibition rate (IR) was calculated using the following formula:

 IR (%) = (1 − mean absorbance of treated tumor/weight/mean absorbance of control tumor/weight) × 100.

## 3. Results

### 3.1. Changes in Histological Findings after Treatment in the HDRA

**Figure 1:**
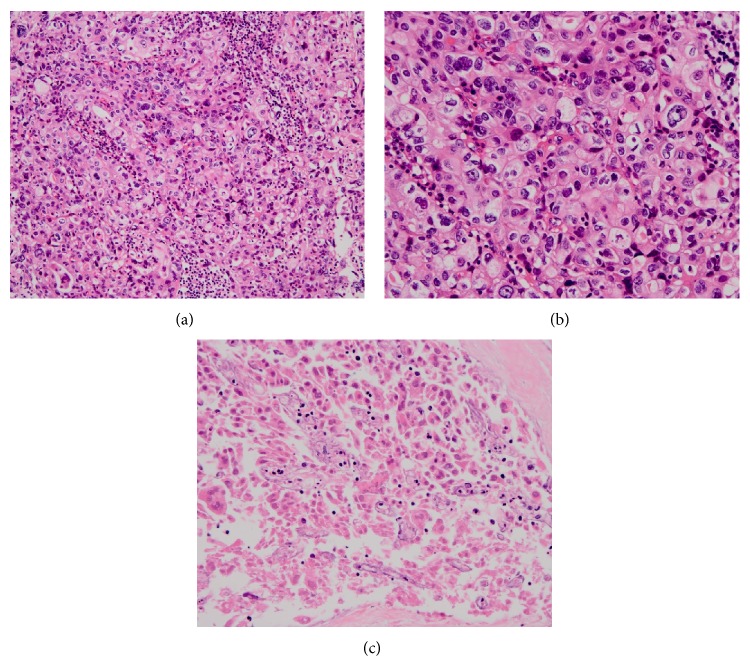
Histopathological structure of a specimen cultured for seven days. ((a) and (b)) Control specimen and (c) specimen exposed to paclitaxel (40 *μ*g/dL).

Figure 1 shows the histopathological structure of a specimen cultured for 7 days.

The structure was sufficiently retained in the control specimen for 7 days, while in the specimen exposed to PTX (40 *μ*g/mL), viable cells were markedly decreased, reflecting the tissue damage and necrosis caused by PTX.

### 3.2. IRs of 10 Different Agents in the HRDA

**Figure 2:**
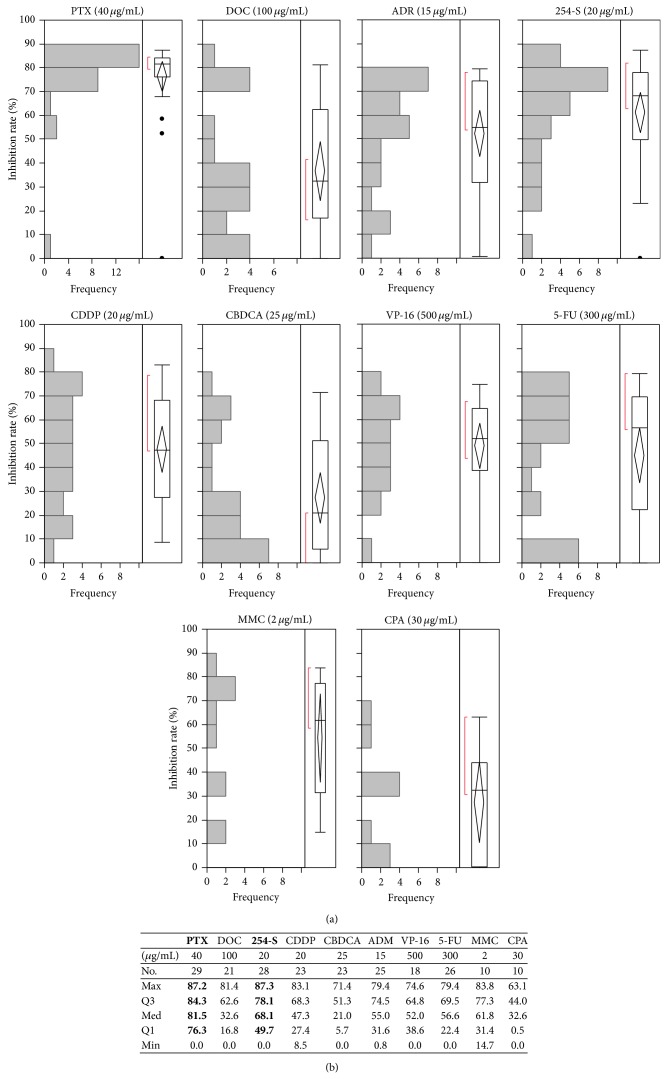
Distribution of inhibition rates using the HDRA in human ATC. PTX: paclitaxel, DOC: docetaxel, 254-S: nedaplatin, CDDP: cisplatin, CBDCA: carboplatin, ADM: doxorubicin, VP-16: etoposide, 5-FU: fluorouracil, MMC: mitomycin C, and CPA: cyclophosphamide. No.: number of cases, Max: maximum IR (%), Q3: third quartile IR (%), Med: median IR (%), Q1: first quartile IR (%), and Min: minimum IR (%).

The distribution of IRs using the HDRA in human ATC is shown in Figure 2. Of these 10 agents, PTX was the most effective agent, and 25 of 29 cases (86.2%) had high IRs over 70% (median 81.5%), while DOC, another taxane, had relatively lower inhibition rates (median 32.6%). 254-S had higher inhibition rates (median 68.1%) than other platins, CDDP (median 47.3%) and CBDCA (median 27.4%).

### 3.3. IRs of Different Doses of PTX and 254-S

**Figure 3:**
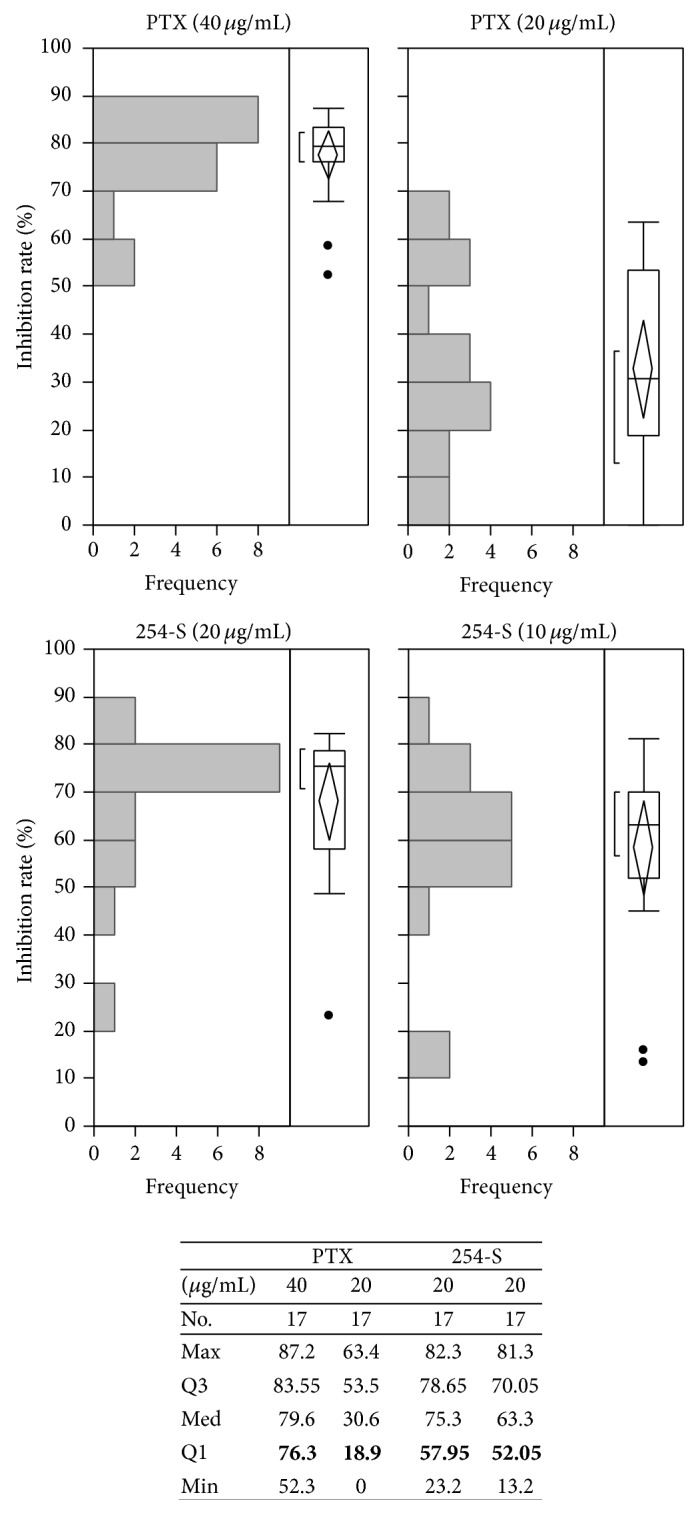
Differences in the inhibition rates between 100% and 50% doses of PTX and 254-S. PTX: paclitaxel, 254-S: nedaplatin. No.: number of cases, Max: maximum IR (%), Q3: third quartile IR (%), Med: median (%), Q1: first quartile IR (%), and Min: minimum IR (%).

Because the concentrations of each drug were determined based on experience with colon, gastric, lung, and breast cancers, it is possible that the dose settings of PTX and 254-S were too high to evaluate the IRs of these agents in ATC. Therefore, 17 samples were tested with both 100% and 50% doses of PTX and 254-S at the same time (Figure 3). The IR of 50% dose PTX (20 *μ*g/mL) was markedly decreased, while that of 50% dose 254-S (10 *μ*g/mL) still maintained its inhibition effect compared to 100% dose.

### 3.4. IRs of Different Sites from the Same Patients

**Figure 4:**
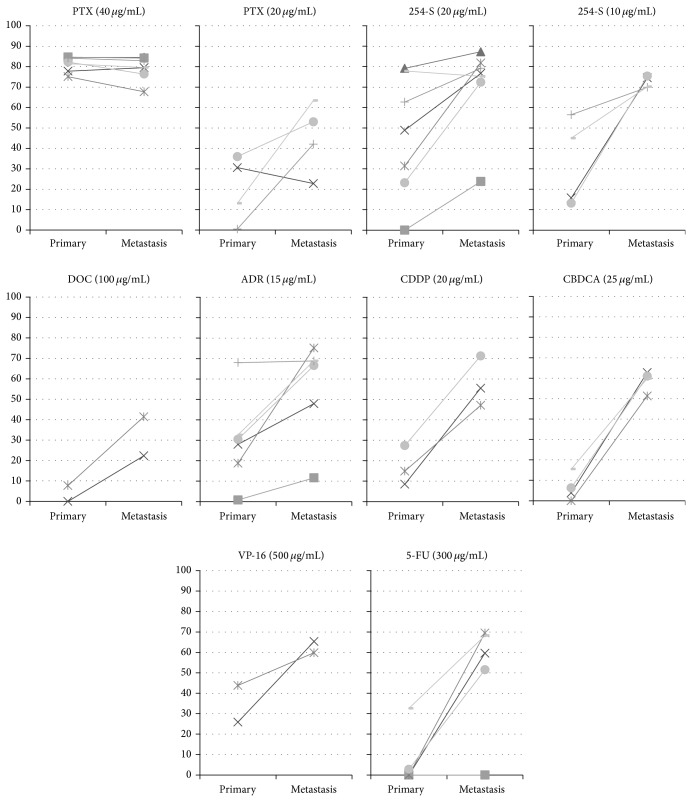
Differences in the inhibition rates between the primary and metastatic lesions from the same patients.

Most recurrent samples had higher inhibition rates than primary lesions, but the tendencies of the IRs for different drugs between primary and recurrent lesions were different even in samples from the same patients (Figure 4).

### 3.5. Correlation between the IR and the Clinical Response to Weekly PTX

A summary of 22 cases is shown in Table 1. In our institute, we usually consider induction chemotherapy with weekly PTX (80 mg/m^2^) before surgery for patients with ATC. In the present case series, 13 of 22 patients (59%) received this neoadjuvant chemotherapy. Therefore, there are some possible biases in the results of the HDRA. The most important bias is that only selected patients who had responded to the induction PTX underwent surgery. Higher IRs of PTX might be reasonable in these patients. However, in contrast, 6 recurrent samples after failure of weekly paclitaxel still had relatively high IRs with 40 *μ*g/mL PTX (median 78.6%, 67.7–84.5%), although these lesions were progressive disease (PD) with paclitaxel treatment. It is difficult to identify a correlation between the IR of 40 *μ*g/mL PTX and the clinical response to weekly PTX (80 mg/m^2^) or the clinical outcome.

## 4. Discussion

The HDRA is one of the chemosensitivity tests for anticancer agents. In vitro assays, the SDI (succinate dehydrogenase inhibition test), HTCA (human tumor clonogenic assay), the HDRA, and the CD-DST (collagen gel drop drug sensitivity test), are examples of anticancer drug sensitivity tests [7]. The advantages of the HDRA are that a high evaluation rate permits the prompt acquisition of results, it correlates with the clinical response, it tests multiple anticancer drugs, and it is comparatively inexpensive [7]. This is thought to be due to the advantages of histoculture methods over other methods using cell suspensions. Histoculture methods maintain cell-to-cell contacts, resulting in good cell viability [9, 15]. The weakness of the HDRA is that some surgical procedure is necessary to obtain sufficient specimens for the HDRA even for inoperable cases, because needle biopsy specimens are not applicable for the HDRA. Even then, the HDRA is quite useful not only to identify personalized medicines for patients with aggressive cancers, but also to find new targeted drugs for malignancies resistant to conventional anticancer treatments.

ATC remains one of the most aggressive and lethal cancers. There are no systemic therapies of proven benefit in terms of improved survival in advanced ATC. Even in patients with stage IVA (intrathyroidal disease) and patients with a small incidental ATC component in resected differentiated thyroid cancer tissues, distant metastases, as well as locoregional recurrences, occur frequently after surgical curative resection [2, 16, 17]. For this reason, most patients with ATC have systemic disease. Thus, to find effective systemic chemotherapy regimens is most important to improve survival after the diagnosis of ATC. For patients with advanced differentiated thyroid cancers, new targeted therapies, such as sorafenib, selumetinib, pazopanib, and sunitinib, have been investigated with promising results, but for patients with ATC, no targeted therapy has been proven to be effective in vivo [18].

In the present study, PTX had the highest IRs to ATC tissues in the HDRA. Some previous reports suggested the usefulness of PTX for the treatment of patients with ATC. A phase II clinical trial of PTX as a 96-hour infusion achieved a favorable outcome; 19 evaluable patients demonstrated a 53% overall response rate (ORR), with 1 (5%) complete response (CR) and 9 partial responses (PR) [19]. Furthermore, long-term survivors were reported among patients with stages IVB and IVC given weekly PTX as induction chemotherapy [20]. In our hospital, induction weekly PTX (80 mg/m^2^) given 4 to 8 times followed by surgical resection is the standard treatment for stages IVA and IVB. After the surgery, additional administration (total 12 to 16 times) followed by whole neck irradiation (40 to 60 Gy) is usually performed. Consequently, many samples for the HDRA were pretreated by PTX (13 of 22 cases, 59%). Because most patients with progressive disease (PD) with PTX treatment or stage IVC usually did not undergo surgery, the samples after PTX administration showed clinical responses of stable disease (SD) to partial response (PR). That might be the reason for the higher IR of PTX. The dose of PTX (40 *μ*g/mL) in the HDRA was determined based on experience with lung and breast cancers. Six recurrent cases after failure of weekly PTX still had relatively high IRs with 40 *μ*g/mL PTX (median 78.6%, 67.7–84.5%), although these lesions were progressive disease (PD) with PTX treatment. Because the 50% dose (20 *μ*g/mL) PTX showed a markedly decreased IR, this dose may not be suitable for ATC and other doses, such as 75% dose (30 *μ*g/mL), will be necessary for future studies. According to reports of uterine cervical cancer [21], 75 *μ*g/mL PTX in the HDRA gives an IR of only 33.8% ± 17.2%. Compared with this report, 40 *μ*g/mL PTX is not an excessively high concentration. In addition, depending on the dose-response curve of PTX in breast cancer, the ED50 was 36.8 ± 17.2 *μ*g/mL [15]. In lung cancer treated by 40 *μ*g/mL PTX, the mean IR was 69.3% ± 22.8%, and 63.2% of cases had IRs over 70% [9]. Indeed, the establishment of the optimal dose of drugs in the HDRA is quite difficult, because the drug transfer rate to cancer tissue is different among organs and cancer sites. Although weekly PTX is more effective than other cytotoxic agents such as anthracyclines and platins, time to progression (TTP) is still around 2–4 months in our experience, and most patients with ATC have short survivals. Therefore, it is also quite difficult to determine the cut-off level of IR for ATC. In the present study, 19 of 22 cases received PTX treatment. Although we analyzed the patients' outcomes based on the IRs of PTX at 80%, 75%, and 70%, no significant differences on PFS or OS were found between the groups of higher and lower IRs (data were not shown). That is the reason why we could not determine the cut-off levels of IRs which are useful to predict the PTX responsiveness.

Another issue is that the cancer cells may be resistant to the drug at the beginning of treatment, or they may become resistant after being exposed to the drug. Resistance can emerge due to a variety of reasons, including host environmental factors, as well as genetic or epigenetic alterations in the cancer cells [22]. Although it may be possible to recreate tumor genetic or epigenetic conditions in the HDRA, the tumor microenvironments associated with drug resistance cannot be estimated under histoculture conditions. The result in the present study that the IR of PTX in recurrent samples after PTX failure was still relatively high might suggest that acquired PTX resistance in patients with ATC is caused by changes in host environmental factors, including tumor microenvironment, rather than cancer cell factors, such as genetic or epigenetic factors. Further studies are necessary to identify these molecular factors related to sensitivity or resistance of ATC to PTX.

However, under the present conditions and limitations in vitro, the higher IR of PTX compared to other common cytotoxic agents in the present study suggests that paclitaxel is the most promising key drug for the treatment of ATC. In our preliminary data, induction PTX resulted in a high rate of curative surgery (16/19 cases, 84.2%), which would also have contributed to longer CSS in stage IVB. In stage IVC (*n* = 56), both PTX (OR, 0.42; 95% CI, 0.08–0.80; *P* = 0.014) and Rx (OR, 0.40; 95% CI, 0.09–0.71; *P* = 0.01) correlated significantly with longer CSS.

Cisplatin (CDDP) and carboplatin (CBDCA) are two major platins and are promising for many kinds of cancers. In Japan, nedaplatin (254-S) is also the agent of choice for cervical cancer and head and neck cancers. It has fewer gastrointestinal side effects and less nephrotoxicity [7]. In the present study, nedaplatin (254-S) was the drug to which ATC showed the second highest sensitivity, and the 50% dose of this drug still inhibited growth of ATC tissues. Further study will be necessary with respect to the clinical use of nedaplatin (254-S) in patients with ATC.

In conclusion, paclitaxel has higher IRs to ATC tissues in the HDRA, and it may be a promising key drug to treat patients with ATC.

## Figures and Tables

**Table 1:** Summary of 22 cases of ATC.

Cases	Age (y)/sex	Site	Curability	Induction PTX	HDRA (1) (%)	Adjuvant PTX	HDRA (2) (%)	PFS	Survival	OS
1	70/F	Primary	Curative	No	83.9	9	NA	3 M (local)	Died	6 M
2	68/F	Primary	Curative	No	0	7 (PD)	NA	1 M (lung)	Died	7 M
3	59/M	Local LN	Curative	No	79.4	16	NA	>52 M (DF)	Alive	>52 M
4	83/F	Primary	Curative	3 (SD)	84.5	12	84.5 (lung)	5 M (lung)	Died	32 M
5	80/M	Primary	Noncurative	5 (SD)	84.3	12 (CR)	NA	9 M (brain)	Died	14 M
6	71/F	Primary	Curative	6 (SD)	71.1	5 (PD)	NA	3 M (local)	Died	4 M
7	75/F	Chest wall	Noncurative	No	81.4	No	NA	0 M	Died	0 M
8	65/F	Primary	Curative	No	83.0	16	NA	10 M (lung)	Died	16 M
9	70/F	Axillary LN	Noncurative	No	84.7	No	84.2 (local LN)	3 M (local)	Died	8 M
10	82/F	Primary	Curative	7 (SD-PR)	52.3	6 (PD)	NA	5 M (local, lung)	Died	7 M
11	59/M	Primary	Curative	8 (PR)	84.7	8 (PD)	NA	5 M (local, lung)	Died	14 M
12	71/M	Primary	Curative	7 (SD-PR)	77.8	7 (PD)	79.5 (local LN)	3 M (local, lung)	Died	7 M
13	62/F	Local rec.	Noncurative	No	75.1	12 (PD)	67.7 (local rec.)	5 M (local)	Died	11 M
14	86/M	Primary	Noncurative	No	77.9	No	NA	1 M (bone)	Died	12 M
15	63/F	Local rec.	Noncurative	4 (PD)	82.3	17 (SD-PD)	76.4 (local rec.)	3 M (local, lung)	Died	12 M
16	59/F	Primary	Noncurative	6 (PR)	84.2	10 (PD)	82.9 (lung)	4 M (lung)	Died	7 M
17	81/F	Primary	Curative	8 (PR)	87.2	4	NA	>17 M (DF)	Alive	>17 M
18	76/F	Primary	Curative	6 (SD)	81.5	7	79.6 (local rec.)	3 M (local)	Died	4 M
19	63/F	Primary	Curative	5 (CR)	58.4	7	NA	>16 M (DF)	Alive	>16 M
20	47/F	Primary	Curative	10 (SD)	76.2	6	NA	>9 M (DF)	Alive	>9 M
21	71/F	Primary	Curative	8 (SD-PR)	87.2	3 (PD)	NA	4 M (local)	Died	4 M
22	79/F	Primary	Curative	No	82.4	12	NA	6 M (lung)	Alive	>7 M

Curability: surgical curability of operation, induction PTX: preoperative treatment by weekly paclitaxel, adjuvant PTX: postoperative treatment by weekly paclitaxel, HDRA (1): induction rates of the samples at the initial surgery with 40 *μ*g/mL PTX, HDRA (2): induction rates of relapsed lesions with 40 *μ*g/mL PTX, OS: overall survival, LN: lymph node, rec.: recurrence, DF: disease-free, CR: complete response, PR: partial response, SD: stable disease, PD: progressive disease, F: female, and M: male.
